# Targeted Molecular Imaging Using Aptamers in Cancer

**DOI:** 10.3390/ph11030071

**Published:** 2018-07-19

**Authors:** Sorah Yoon, John J. Rossi

**Affiliations:** 1 Department of Molecular and Cellular Biology, Beckman Research Institute of City of Hope, Duarte, CA 91010, USA; 2 Irell and Manella Graduate School of Biological Sciences, Beckman Research Institute of City of Hope, Duarte, CA 91010, USA

**Keywords:** aptamer, targeted imaging, live cells, tissue, in vivo, cancer

## Abstract

Imaging is not only seeing, but also believing. For targeted imaging modalities, nucleic acid aptamers have features such as superior recognition of structural epitopes and quick uptake in target cells. This explains the emergence of an evolved new class of aptamers into a wide spectrum of imaging applications over the last decade. Genetically encoded biosensors tagged with fluorescent RNA aptamers have been developed as intracellular imaging tools to understand cellular signaling and physiology in live cells. Cancer-specific aptamers labeled with fluorescence have been used for assessment of clinical tissue specimens. Aptamers conjugated with gold nanoparticles have been employed to develop innovative mass spectrometry tissue imaging. Also, use of chemically conjugated cancer-specific aptamers as probes for non-invasive and high-resolution imaging has been transformative for in vivo imaging in multiple cancers.

## 1. Introduction

Recent decades have seen remarkable progress in molecular imaging, which is an indispensable tool for bench to bedside applications [1,2]. The array of imaging techniques currently available include optical imaging (fluorescence and bioluminescence), magnetic resonance imaging (MRI), positron-emission tomography (PET), single-photon emission computed tomography (SPECT), computed tomography (CT), and ultrasound (US), which are used in detection and characterization of cancers and assessment of responses for therapeutic intervention. To achieve satisfactory imaging, many factors have to be considered; suitable probes that have low target-to-background-noise signals, low toxicity, and suitable stability are indispensable [3]. To achieve this, antibodies or their derivatives targeted to specific antigens are typically employed [4]. However, in terms of target specificity and stability for imaging probes, aptamers are better tools, with greater advantages over antibodies, including target-specific recognition, high target-binding affinity, low immunogenicity, structural stability, and comparably smaller size (~3 nm vs. 10–15 nm for antibodies) [5]. Their excellent functions as imaging probes are demonstrated by super-resolution microscopy, showing superior and brighter targeted imaging of membrane receptors and intracellular organelles than antibodies [6]. In a comparison study of ^111^Indium (In)-labeled aptamers with ^111^In-antibodies using SPECT/CT images, ^111^In-labeled aptamers showed better tumor-specific uptake than ^111^In-antibodies after intravenous injection at 4 h [7], because of their smaller size relative to antibodies. As these two studies clearly show, aptamers have improved function over antibodies, and they have quickly emerged as molecular imaging probes for both subcellular and in vivo applications. Thus, in this review, we focus on several types of aptamer-guided imaging techniques based on recently released research, covering subcellular imaging to in vivo imaging in cancer.

## 2. Cell Imaging

Cell imaging is considered essential for understanding cellular physiology. Historically, green fluorescent protein (GFP) has been central to cellular imaging applications by way of being tagged onto endogenously coding genes to investigate the expression, interaction, localization, and role of target proteins in cells [8,9]. However, the vast amount of research in the RNA field has started to expand the role of RNAs, leading to development of innovative tools for intracellular imaging. This includes fluorogenic aptamers that activate fluorescence upon binding of their target fluorophores; e.g., Spinach [10], Broccoli [11], Mango [12], and Corn [13]. For in vitro applications, these fluorogenic aptamers can be engineered as biosensors to detect intracellular metabolites or imaging tags to investigate the dynamics of intracellular RNAs in living cells by microscopy. To date, no in vivo imaging has been conducted with these fluorogenic aptamers. However, these fluorogenic aptamers have great potential for in vivo imaging. Thus, in this section, we will discuss fluorogenic aptamers and their in vitro applications.

### 2.1. Green Fluorogenic Aptamers: Spinach and Broccoli

The “Spinach” fluorogenic RNA aptamer activates green fluorescence upon the binding of the small-molecule fluorophore 3,5-difluoro-4-hydroxybenzylidene imidazolinone (DFHBI) and its variants (e.g., DFHBI 1T and DFHBI 2T) [10]. To investigate the precise working mechanism of fluorescence activation, the Spinach-DFHBI complex was determined by analysis of the crystal structures. The co-crystal structures of the Spinach-DFHBI complex revealed an unexpected quadruplex core connected with two G-tetrads and a mixed-base tetrad [14]. DFHBI was inserted by a base triple and confined around an unpaired guanine residue (G31) [14]. In comparison with fluorophore-free condition, the collapsed fluorophore binding site was rearranged with the base tier of the quadruplex, extruding the G31 from the helical stack [14]. The fluorogenic Spinach was a novel proof-of-concept study, but weaknesses of Spinach such as thermal instability, requirement of high magnesium concentration, misfolding, and reduced brightness [15] have limited its application for imaging. In the following studies, Spinach was mutated to multiple variants such as Spinach2 [15] and Baby Spinach [16] to increase thermal stability and folding structure.

As Spinach had some drawbacks, the microfluidic-assisted In Vitro Compartmentalization (μIVC) method was employed to screen for aptamers able to work in low bivalent concentration and the selected aptamers were minimized for in vitro application, which was named iSpinach [17]. In a comparison of iSpinach with Spinach2, iSpinach showed 1.4 times more fluorescence in the presence of potassium, better thermostability, and binding affinity than Spinach2. However, live-cell imaging was not evaluated.

For live-cell imaging, Spinach and Spinach2 were engineered as biosensors to detect cellular metabolites (S-adenosylmethionine (SAM), adenosine 5-diphosphate (ADP), and second messenger Cyclic di-AMP (cdiA)) in *Escherichia coli* (*E. coli*) and *Listeria monocytogenes* (*L. monocytogenes*) [18,19]. Spinach engineered with SAM-binding RNA aptamers via a stem sequence that acted like a transducer for a biosensor showed 6-fold increased fluorescence over 3 h, when the SAM precursor methionine was provided in DFHBI-treated *E. coli* [18]. The Spinach-ADP sensor showed a 20-fold increase of fluorescence in DFHBI-treated *E. coli* [18]. For imaging of intracellular cdiA levels in *L. monocytogenes*, the ligand-sensing domain of cdiA riboswitch (yuaA riboswitch) was combined with Spinach2, termed YuaA-Spinach2, to act as fluorescent biosensors [19]. When a plasmid encoding the biosensor was transformed into live *L. monocytogenes*, robust intracellular fluorescence was observed under fluorescence microscopy in the presence of DFHBI [19]. This clearly demonstrates that the yuaA-Spinach2 biosensor works in living cells.

To overcome the limitations of Spinach, the green fluorogenic RNA aptamer “Broccoli”, which binds to the fluorophore DFHBI, was isolated using a different approach by the same group that developed Spinach [11]. The method of Broccoli isolation was designed to improve the specificity and functionality of the fluorogenic aptamer. In experimental approaches, fluorescence RNA aptamers when treated with fluorophores after first round of conventional aptamer selection method were inserted into plasmid. After transformation of cloned plasmids into *E. coli.*, fluorescence cells were isolated by fluorescence-activated cell sorting (FACS) method in the treatment of DFHBI. This approach successfully isolated the fluorogenic aptamer Broccoli, which showed robust folding in low salt concentrations, increased thermostability, and stronger fluorescence relative to Spinach2 [11]. For further in vitro imaging, Broccoli was truncated to tBroccoli and was dimerized to tdBroccoli. The dimerized tdBroccoli showed twice the fluorescence intensity compared to Broccoli in living *E. coli*. [11]. To make a biosensor, Broccoli, tBroccoli or tdBroccoli was fused to the 3′ end of 5S plasmid with or without the pAV5S tRNA scaffold. Fluorescence activation was compared in transfected HEK293T cells using microscopy. The Broccoli or dBroccoli without a tRNA scaffold showed higher fluorescence intensity than that with the tRNA scaffold [11]. What the Broccoli study suggests is that the strong thermostability and folding structure of aptamers do not require a tRNA scaffold system to support the appropriate folding of aptamers intracellularly, which is a valuable advantage for in vivo imaging in the future.

### 2.2. Orange Fluorogenic Aptamer: Mango

The “Mango” RNA aptamer binds to derivatives of the fluorophore thiazole orange (TO1) and shows high levels of orange fluorescence, which increase by up to 1100-fold upon the binding of TO1 [12]. The structure of Mango was predicted to be folded with two-tiered all-parallel G-quadruplexes in an A-form duplex [12]. Crystal structure analysis of the Mango-TO1 complex showed three antiparallel quinines forming G-quadruples [20]. The binding of TO1 on one of the flat faces of the G-quadruplex stabilized the Mango structure and activated orange fluorescence. For imaging applications, TO1 was directly injected into *Caenorhabditis elegans* (*C. elegans*) syncytial gonads with equimolar amounts of RNA Mango. The strong fluorescence composition was observed in the nuclei of gonad [12].

New orange fluorogenic RNA Mango aptamers were isolated by the group that isolated iSpinach using microfluidics-based selection methods [21]. This “New Mango” was brighter than enhanced green fluorescent protein (EGFP) when bound to TO1, which is a major advantage for imaging applications. For imaging sensors, New Mango was tagged by incorporating the human 5S ribosomal RNA or U6 small nuclear RNA (snRNA) into F30 folding scaffold plasmids. The RNAs of 5S-F30-New Mango or U6-F30-New Mango generated by in vitro transcription were transfected into mammalian HEK293T [21]. Subcellular co-localization and fluorescence were investigated using fluorescence microscopy. The 5S-F30-New Mango RNAs showed up to 10 bright RNA foci per cell in the cytoplasm, mainly co-localized with mitochondria [21]. The U6-F30-New Mango RNA showed a different localization; it was mainly co-localized with small nuclear protein (snRNP) Lsm3 [21]. To develop a genetically encoded imaging tag expressed in mammalian cells, New Mango was inserted into the pSLQ plasmid to express 5S rRNA under the RNA pol III promoter (pSLQ-5S-F30-New Mango) [21]. The engineered pSLQ-5S-F30-New Mango plasmid was transfected into cells. The expression of fluorescence was observed to be both nucleolar and cytoplasmic using fluorescence microscopy. pSLQ-5S-F30-New Mango was also observed to co-localize with ribosomal protein L7 [21]. These results clearly demonstrate that New Mango has the capability to be used as an imaging tag to visualize the subcellular location of target genes.

### 2.3. Yellow Fluorogenic Aptamer: Corn

The RNA aptamer that binds to 3,5-difluoro-4-hydroxynenzylidene imidazolinone-2-oxime (DFHO), named “Corn”, activates yellow fluorescence [13]. Compared with Broccoli, Corn showed strong photostability; its fluorescence was detected at 320-ms imaging times vs. 160 ms with Broccoli [13]. In irradiation experiments, the Corn–DFHO complex exhibited minimal loss of fluorescence after as long as 10 s vs. >50% of fluorescence was lost after 200 ms with the Broccoli–DFHBI complex. Therefore, Corn was considered an emerging new fluorogenic aptamer for biosensors. To test the feasibility of Corn as a genetically encoded fluorescent RNA tag, three main subclasses of Pol III promoters (5S RNA, tRNA, and U6) with a tRNALys scaffold plasmid were constructed with the Corn aptamer. When these reporter plasmids were transfected into HEK293T cells, yellow fluorescence was observed by fluorescence microscopy after treatment with DFHO [13].

### 2.4. Rainbow Fluorogenic Aptamer: SRB-2

The SRB-2 aptamer, originally isolated against the fluorophore sulforhodamine B, binds to various dyes with xanthene-like cores, such as pyrosin B, pyrosin Y, acridine orange, SR-DN, and TMR-DN, and yields a “rainbow” of differently colored bright fluorescence [22]. To confirm different fluorescence by the SRB-2 aptamer upon binding of different fluorophores, a single copy of the SRB-2 aptamer sequence was inserted into tRNA scaffold plasmid. After transformation of SRB-2 inserted plasmid into *E. coli*, the activation of fluorescence was investigated using microscopy in the presence of a variety of fluorophores. The SRB2-expressing bacteria showed various colors after incubation with each fluorophore: pyrosin B (yellow color), pyrosin Y (yellow color), acridine orange (green color), SR-DN (red color), and TMR-DN (yellowish-orange color) [22]. As the SRB-2 aptamer showed various colors of fluorescence, selection of an appropriate fluorophore was investigated for subsequent no-wash and live cell imaging methods. Even though pyrosin B, pyrosin Y, and acridine orange showed differently colored fluorescence, the three fluorophores showed high signal-to-background ratio, compared with SR-DN and TMR-DN. SR-DN was previously developed as a live-cell imaging fluorophore for the SRB-2 aptamer, but it had low binding affinity (K_d_ = 1.3 μM). In contrast, TMR-DN showed higher binding affinity (K_d_ = 35 nM) for the SRB-2 aptamer. In a comparison of SR-DN and TMR-DN, SRB-2 expressing bacteria displayed 11-fold higher fluorescence intensity after incubation with TMR-DN than with SR-DN [22]. Therefore, TMR-DN was used for subsequent live cell imaging. For live-cell imaging of rRNA and mRNA in mammalian cells, 5S rRNA embedded with a single repeat of SRB-2 in a tRNA scaffold was applied in mammalian cell imaging [22]. The nuclear and cytoplasmic distribution of fluorescent 5S rRNA were clearly visualized using a 5S-SRB-2 construct in HeLa cells by microscopy [22]. For mRNA imaging in live prokaryotic cells, 6 and 15 tandem repeats of SRB-2 without a tRNA scaffold were fused to the 3′-UTR GFP mRNA. Bacteria expressing GFP-6xSRB-2 and GFP-15xSRB-2 showed significantly higher fluorescence than bacteria expressing only GFP after incubation with TMR-DN. For mRNA imaging in live eukaryotic cells, 15 repeats of SRB-2 without a tRNA scaffold were fused to 3′-UTR CFP-TM (cyan fluorescent protein). Microscopy revealed that HeLa cells transfected with CFP-TM-15xSBR-2 plasmid showed significantly higher fluorescence in TMR-DN than control cells, with mostly cytoplasmic distribution of the CFP-TM mRNAs [22].

**Figure 1:**
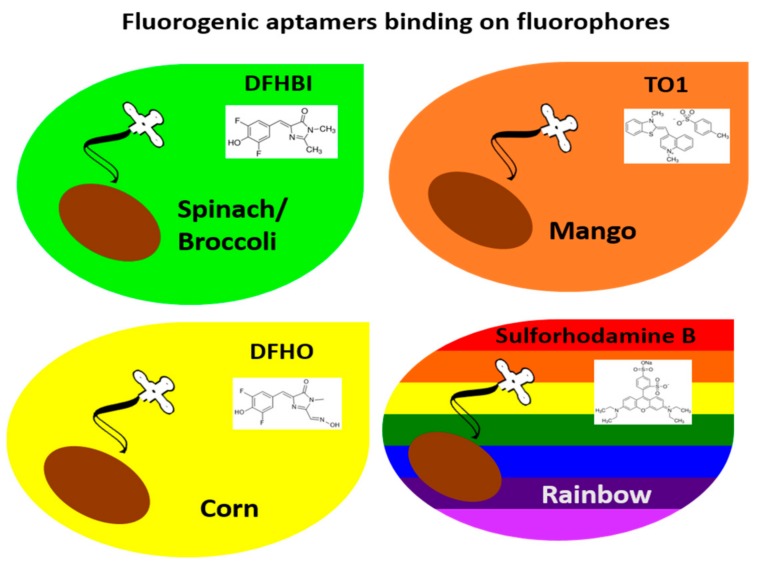
Live cell imaging with bioengineered aptamer tags. Fluorogenic RNA aptamers such as Mango, Corn, Broccoli, and SRB-2 Rainbow are inserted after a gene of interest, bind to their target fluorophores, and turn on fluorescence in the presence of their target fluorophores. The fluorogenic RNA aptamer tags can be used for tracking and for investigating the dynamics of a gene of interest in live cells.

Taken together, fluorogenic RNA aptamers such as Spinach, Broccoli, Mango, Corn, and Rainbow aptamers can be successfully applied for intracellular imaging in live cells. Thus, these fluorogenic RNA aptamers are great tools to investigate the dynamics of intracellular RNAs, as depicted in Figure 1. Theoretically, RNA aptamers can be developed to any fluorophores, and because fluorogenic RNA aptamer tags have diverse applications, we expect a variety of fluorogenic aptamers suitable for mammalian in vivo imaging will be developed in the near future.

## 3. Tissue Imaging

Tissue imaging using aptamers remains an underdeveloped research area. Because aptamers have high target specificity and cancer-specific aptamers are being continuously discovered [23], the assessment of clinical tissue specimens with labeled aptamers allows us to characterize tumors. However, to date, there are few studies investigating the use of fluorescently labeled or gold metal-conjugated aptamers to assess cancer characteristics.

The pancreatic cancer specificity of RNA aptamers isolated via cell-SELEX was assessed using Cy3-labeled RNA aptamers on human pancreatic cancer patient specimens and human normal tissues [24]. The pancreatic-cancer-specific RNA aptamers showed strong fluorescence intensity that correlated with the low survival rate of pancreatic cancer patients [24]. This implies that the target ligand of the pancreatic cancer-specific aptamer could be used a prognostic marker, when the aptamer-binding target is identified.

The DNA aptamer DML-7, which shows specific binding to metastatic prostate cancer DU145 cells, was evaluated on normal, non-metastatic, and metastatic prostate cancer (PC) tissue specimens with Cy5-labeled aptamers [25]. No fluorescence was observed on normal prostate tissues and weak fluorescence was observed on non-metastatic prostate tissues. However, moderate to strong fluorescence was observed on metastatic PC tissues [25]. This suggests that DML-7 aptamers bind to a cancer metastatic biomarker.

**Figure 2:**
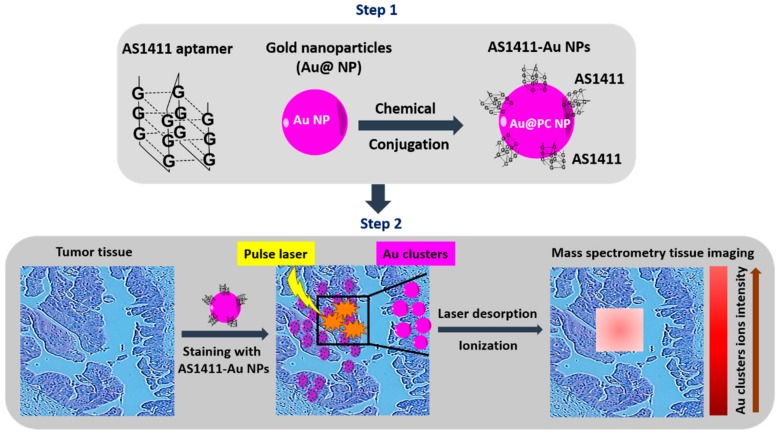
Mass spectrometry tissue imaging (MSI). Anti-nucleolin aptamers conjugated with gold nanoparticles are used to stain cancer tissues. Under laser desorption/ionization, gold cluster ion-amplified signal reporters are collected for laser MSI.

Mass spectrometry tissue imaging (MSI) is a very attractive imaging technique for analysis of multiple biomolecules simultaneously and biomarker discovery from tissue samples [26]. Recently, laser desorption/ionization MS (LDI-MS) techniques has been developed to analyze the distribution of nanomaterials on tissue specimens [27]. As a proof-of-concept study for tissue MSI, the anti-nucleolin aptamer AS1411 was conjugated with gold nanoparticles (GNPs) [28]. Under laser desorption/ionization, gold cluster ions (Aus) were disassociated from AS1411-gold nanoparticles and acted as signal amplifiers to detect nucleolin-positive tumor tissue images. The aptamer-guided MSI imaging was tested on human breast tumor and normal breast tissues. The tissue samples were labeled with AS1411-gold nanoparticles and LDI-MS imaging was acquired in positive-ion mode using an Autoflex III LDI TOF mass spectrometer. A SmartBeam laser (355 nm Nd: YAG) was operated at 100 Hz to irradiate the tissue slides, using conditions of laser spot diameter at 30 μm and pixel step size at 150 μm [28]. The MS images generated through monitoring of Au signal intensity clearly indicated that the tumor tissues had much stronger signal intensities than normal tissues. Furthermore, the expression and distribution of nucleolin in breast tissue by immunohistochemistry (IHC) showed consistent results with those obtained through LDI-MS. This successful imaging acquisition using aptamers has major implications for aptamers as suitable imaging probes in an MSI platform for the diagnosis of malignancies. The schematic work flow is depicted in Figure 2. However, a disadvantage of LDI-MS imaging is that it can only detect highly abundant targets on tissue samples. Therefore, it provides more valuable diagnostic information when combined with in vivo imaging.

## 4. In Vivo Imaging

Identifying target-specific probes is critical to conferring specificity for in vivo imaging. In this respect, the majority of aptamers that have already been developed target cancer-specific surface antigens [23]. Therefore, these cancer-specific aptamers are great imaging probes for a variety of imaging techniques in cancer diagnostics, including fluorescence optical imaging, MRI, PET, SPECT, CT, US, and photoacoustic imaging. In this section, we will discuss cancer-specific aptamers labeled with multiple imaging dyes for targeted in vivo imaging.

### 4.1. Fluorescence Imaging

Fluorescence is the most adapted tool in aptamer-guided imaging because of its low cost and high sensitivity. The most commonly used fluorescent dyes are cyanine 5 (Cy 5), quantum dots (QDs), and near-infrared (NIR) dye.

Cancer cell-type-specific DNA aptamers against Ramos cells (B-cell lymphoma) were labeled with a Cy5 probe (Cy5-TD05) and the efficacy of in vivo imaging was determined in subcutaneously xenografted nude mice via tail vein injection [29]. This demonstrated that Cy5-TD05 was specifically accumulated into engrafted tumor regions by 3.5 h or later, compared with control aptamers, Cy5 dye only, or in non-targeted cells. Cy5-TD05 also showed a high signal-to background ratio at 115.50. In biodistribution assays, high fluorescence was observed in the small intestine [29]. In another cancer cell-type-specific aptamer study, DNA aptamers specific to A549 cells (lung carcinoma cells) were labeled with a Cy5 probe (Cy5-S6) for in vivo imaging [30]. In subcutaneously xenografted nude mice, Cy5-S6 showed tumor-specific accumulation between 3 to 5 h after intravenous injection. In contrast, no fluorescence was observed with a Cy5-labeled library or in non-targeted cells. Using the same approach, two other cancer cell-type-specific DNA aptamers against Bel-7404 and SMMC-7721 (human hepatocellular carcinomas) were tested in vivo. Both aptamers showed tumor-specific fluorescence accumulation by 3 h post-injection.

QDs have been studied as another fluorescence imaging dye in various cancer models. QDs are fluorescent nanoparticles that have great advantages for imaging, such as low photobleaching, high fluorescence yield, and chemical stability [31]. Fluorescence imaging using QDs has been investigated by chemical conjugation with anti-mucin 1 (MUC1) aptamers and anti-variant of epidermal growth factor receptor (EGFRvIII) aptamers in vivo. As MUC1 is highly expressed on the cancer plasma membrane, the cancer specificity of an anti-MUC1 DNA aptamer conjugated with QDs was evaluated in A549 cells [32]. Anti-MUC1 DNA aptamers labeled with QDs were injected into subcutaneously xenografted nude mice via the tail vein. Strong fluorescence was observed in the tumor specifically, but not with control QDs. EGFRvIII is selectively expressed on cancer cells, and is involved in enhancement of cancer cell proliferation, invasion, and resistance to chemotherapy and radiotherapy [33]. Therefore, it has been popularized as a target for therapeutics and imaging. The anti-EGFRvIII DNA aptamer (A32) was conjugated with streptavidin-PEG-CdSe/ZnS QDs (QD-A32 Apt). For in vivo imaging, U87 (glioma) cells and U87-EGFRvIII overexpressing cells were orthotopically xenografted into nude mice [34]. After tail-vein injection of QD-A32 Apt, fluorescence imaging was performed 6 h post-injection. Mice harboring U87-EGFRvIII overexpressing cells, but not U87 tumors, showed strong fluorescence signals in tumor areas. No significant fluorescence was observed in mice injected with unconjugated QDs. In a biodistribution assay, QD-A32 Apt showed high accumulation in the tumor region, liver, and kidneys, and low accumulation in the tumor pararegion, normal brain, spleen, heart, and lungs. In another study, anti-EGFR aptamers targeting wild-type EGFR were conjugated with lipid QDs (Apt-QLs) [35]. The feasibility of using Apt-QLs for in vivo imaging was tested in an MDA-MB-231 breast in vivo tumor model. By 4 h post-intravenous injection, it showed breast cancer-specific accumulation in engrafted mice [35].

As NIR dyes enhance sensitivity due to low background autofluorescence and higher signal-to-noise ratios, it is another suitable fluorescent dye for in vivo imaging. In PC, anti-prostate-specific membrane antigen (PSMA) RNA aptamer (A9g) was conjugated with NIR dye (IRDye 800CW) [36]. The NIR-labeled aptamer (NIR-A9g) was injected into the tail veins of mice bearing PC-3 (PSMA-positive) tumor cells. It showed intense cancer-specific fluorescence, beginning at 24 h and up to 72 h after administration [36]. In another study, a new NIR dye, which is a derivative of indocyanine green (MPA; absorbance peak at 780nm and emission peak at 810 nm), was tested in breast cancer and liver cancer animal models. Anti-MUC1 DNA aptamers were conjugated with the MPA dye (APT-MPA) or with polyethylene glycol-NIR (APT-PEG-MPA) [37]. MCF-7 breast cancer cells or HepG2 liver cancer cells were subcutaneously xenografted into nude mice. MPA, APT-MPA, or APT-PEG-MPA were injected into mice via tail-vein injection. Strong tumor-specific fluorescence by targeting of APT-MPA or APT-PEG-MPA was observed starting at 2 h up to 72 h on both breast and liver cancers in vivo. In contrast, control MPA did not show accumulation of fluorescence in the tumor-engrafted region [37]. In a biodistribution assay, the PEG-modified probe (APT-PEG-MPA) showed much quicker clearance than that of APT-MPA [37].

### 4.2. Magnetic Resonance Imaging (MRI)

MRI is a very powerful imaging technique used for non-invasive visualization of internal organ structures and soft tissue morphology by using a powerful magnet and radiofrequency energy [38].

Introducing MR imaging contrast agents by way of specific targeting moieties like aptamers has significantly improved the quality of imaging. The α_v_β_3_ integrin subunit is involved in cell migration and invasion during neovascularization and/or angiogenesis in cancer. Therefore, it was targeted for molecular imaging [39]. An anti-α_v_β_3_ aptamer (Apt α_v_β_3_) was conjugated with magnetic nanoparticles (MNPs) for cancer-specific detection via MRI imaging [40]. The aptamer α_v_β_3_-conjugated magnetic nanoparticles (Apt α_v_β_3_-MNPs) were injected into epidermoid carcinoma A431 xenografted nude mice intravenously. The evaluated MR signals showed the maximum intensity at 24 h post-injection. The accumulated amount of Apt α_v_β_3_-MNPs was 129 ± 34.3% in the tumor compared to short peptide conjugated MNPs as control.

Vascular growth factor receptor 2 (VEGFR2) is commonly overexpressed in the tumor vasculature, and could be another target-of-interest for cancer imaging. For the recognition of glioblastoma vasculature, anti-VEGFR2 aptamers were immobilized on the surface of carboxylated magnetic nanocrystals (MNCs) [41]. The molecular imaging potential of the VEGFR2 aptamer-MNCs (VEGFR2 Apt-MNCs) were assessed in an orthotopic glioblastoma nude mouse model. MRI was performed on the animal after intravenous tail veil injection of VEGFR2 Apt-MNC or unconjugated MNC with a 3.0-T MR imaging. At 4 h post-injection, MR imaging was darker in tumor sites treated with VEGFR2 Apt-MNC than in those treated with unconjugated MNC, confirming that VEGFR2 Apt-MNC enabled precise in vivo detection [41].

A common feature of solid cancer is hypoxia, which induces the activation of hypoxia-inducible factor-1α (HIF-1α) [42]. HIF-1α is also involved in the maintenance of cancer stem cells. For non-invasive imaging to identify hypoxia-induced cancer stem cells, HIF-1α aptamer-PEG-modified manganese magnetic nanoparticles, D-Fe_3_O_4_@Mn, were generated [43]. For in vivo imaging, PANC-1 pancreatic cancer cells were subcutaneously xenografted in nude mice. D-Fe_3_O_4_@Mn was injected into the mice intravenously and MRI was performed before and after injection at 2 h. Compared with images of mice without NP, the D-Fe_3_O_4_@Mn-injected mice showed significantly bright enhanced effects and 3.5-fold higher signal intensity in T1-weighted MRI imaging (T1W1). Upon histopathological examination, no signs of toxicity were observed in the major organs at day 10 after injection of D-Fe_3_O_4_@ Mn [43].

### 4.3. Single-Photon Emission Computed Tomography (SPECT)

Nuclear imaging techniques like SPECT and PET can monitor biological events deep within the body and provide longitudinal assessment of a patient with high detection sensitivity [44]. Therefore, development of imaging modalities for SPECT and PET with high specificity is critical. In this respect, cancer-specific aptamers are excellent detecting probes. SPECT imaging detects γ-ray emission from radioisotopes using a gamma camera. The most commonly used isotopes for SPECT imaging are ^99^mTc (Technetium, t_1/2_: 6 h), ^111^In (Indium, t_1/2_: 2.8 d), ^123^I (Iodine, t_1/2_: 13.2 h), and ^131^I (Iodine, t_1/2_: 8.0 d) [45]. For SPECT imaging, an anti-tenascin-C aptamer (TTA1) was conjugated to the succinimidyl ester of MAG2 and radiolabeled with ^99^mTc (MAG2-TTA1-^99^mTc) [46]. To assess in vivo tumor targeting, the MAG2-TTA1-^99^mTc was intravenously injected into mice bearing U215 glioblastoma or MDA-MB-435 breast cancer tumors. After aptamer-based γ-camera imaging of the tumors, the U251 glioblastoma was prominently visible at 3 h, but not after application of control aptamers. The MDA-MB-435 breast tumor showed cancer-specific imaging at 20 h. Biodistribution assays showed that bladder and liver (10 min) and bladder and intestines (3 h) were predominant clearance pathways [44]. When the radiometal chelator was switched to DTPA (DTPA (^111^In)-TTA1), the DTPA (^111^In)-TTA1 showed persistent accumulation in the liver and kidney at 3 h.

EGFR is overexpressed in 90% of head and neck cancers [47]. For comparison studies of aptamers with antibodies, an anti-EGFR aptamer (apt) and antibody (C225) were conjugated with Hollow gold nanospheres (HAuNS), followed by radiolabeling with ^111^In-DTPA. For in vivo imaging, the apt-HAuNS-^111^In-DTPA, C225-HAuNS-^111^In-DTPA, and control-HAuNS-^111^In-DTPA were injected intravenously into OSC-19 (tongue squamous cell carcinoma cell) orthotopic xenografted nude mice. SPECT imaging was performed at 4 h and 24 h post-injection [47]. The imaging revealed that at 24 h post-injection, more apt-HAuNS-^111^In-DTPA accumulated in the tumor than C225-HAuNS-^111^In-DTPA. These results demonstrate that aptamers are easily uptaken into tumors, likely due to their relatively smaller size compared to antibodies, suggesting that aptamers might be better tumor-homing ligands. In a biodistribution assay, apt-HAuNS-^111^In-DTPA showed greater retention in blood, kidneys, and lymph nodes. In contrast, C225-HAuNS-^111^In-DTPA showed greater retention in the liver and spleen.

Human matrix metalloprotease-9 (hMMP-9) is overexpressed in cancers, particularly in cutaneous melanomas [48]. An RNA aptamer against hMMP9 (F3B) was conjugated with ^111^In-DOTA (^111^In-DOTA-F3B). After intravenous injection of the ^111^In-DOTA-F3B into mice bearing human melanoma tumors, SPECT images were acquired at 1 h. The micro-imager analysis showed that ^111^In-DOTA-F3B induced a stronger accumulated signal than ^111^In-DOTA-control. Signal activity was increased in a tumor grade-dependent manner in ex vivo images [49]. The biodistribution of ^111^In-DOTA-F3B was determined at 30 min, 1 h, and 2 h post-injection. The average half-life was 11 min, which represents a rapid clearance from the blood. It also showed high uptake in kidneys and liver, but not other normal organs. The uptake of ^111^In-DOTA-F3B in the tumor region was significantly higher at 1 h post-injection, compared with ^111^In-DOTA-control-aptamer.

A variant of the anti-EGFRvIII DNA aptamer (U2) was labeled with rhenium radioisotope (^188^Re) [50]. The SPECT molecular imaging of ^188^Re-U2 was tested in glioblastoma U87MG xenografted mice at 1 h post-injection. The ^188^Re-U2 aptamer, but not control aptamer, was retained in tumors specifically. Its main route of excretion was through the urinary tract [50].

### 4.4. Positron-Emission Tomography (PET)

PET has at least ten-fold more sensitivity than SPECT [51]. Therefore, it has quickly emerged as a robust molecular imaging technique. For PET imaging probes, four chelators (DOTA, CB-TE2A, S-2-(4-Isothiocyanatobenzyl)-1,4,7,10-tetraazacyclododecane-tetraacetic acid [DOTA-Bn], and S-2-(4-Isothiocyanatobenzyl)-1,4,7-triazacyclononane-1,4,7-triacetic acid [NOTA-Bn]) were selected and used to label the anti-nucleolin AS1411 aptamer, along with ^64^Cu [52]. In comparison studies of these chelators, ^64^Cu-DOTA-AS1411 and ^64^Cu-CB-TE2A-AS1411 were internalized into cells at significantly greater levels than ^64^Cu-DOTA-Bn-AS1411 and ^64^Cu-NOTA-Bn-AS1411. Thus, ^64^Cu-DOTA-AS1411 and ^64^Cu-CB-TE2A-AS1411 were chosen for further in vivo imaging. ^64^Cu-DOTA-AS1411 or ^64^Cu-CB-TE2A-AS1411 was injected into H460 lung cancer-bearing mice via tail-vein injection and mice were imaged at multiple time-points up to 24 h post-injection. In mice injected with ^64^Cu-CB-TE2A-AS1411, tumors were visible from 1–24 h. However, the tumor was not detectable in mice injected with ^64^Cu-DOTA-AS1411. This suggests that CB-TE2A is a more desirable chelator for in vivo kinetics. In biodistribution assay, ^64^Cu-CB-TE2A-AS1411 also had faster clearance and less accumulation in the liver and kidney at 1 h post-injection than ^64^Cu-DOTA-AS1411 [52].

Tenascin C shows a pattern of high expression in tumor stroma and plays a pivotal role in brain and breast cancer initiation and progression [53,54]. Anti-tenascin C DNA aptamers were conjugated with ^64^Cu-NOTA to construct ^64^Cu-NOTA-tenascin C [55]. For targeted molecular imaging of cancer, U86 MG glioma cells and H460 lung cancer cells were subcutaneously xenografted into mice and MDA-MB-435 breast cancer cells were orthotopic xenografted into the mammary glands of mice. The U86 MG and MDA-MB-435 tumors were positive for tenascin C, whereas the H460 was negative. After injection of ^64^Cu-NOTA-tenascin C, PET images were taken at 1, 2, 6 and 24 h in the three cancer cell-engrafted mouse models. Tumor accumulation was clearly evident up to 24 h post-injection in U86 MG and MDA-MB-435 engrafted mice, but not in H460 engrafted mice [55].

Conventionally, aptamers were directly conjugated with a radioactive tracer isotope for PET imaging. As a more versatile platform for PET imaging probes, a hybridization strategy between two complementary oligonucleotides (ODN) has recently been used to label aptamers. The advantage of this platform is that the radioactive tracer isotope can be easily switched. As a proof of concept, AS1411 aptamers were hybridized with fluorine radioisotope (^18^F)-labeled complementary ODN9 (cODN) [56]. The feasibility of hybridization-based aptamer labeling for PET imaging was tested in vivo. C6 glioma-bearing mice were injected with ^18^F-hyAS1411 and ^18^F-hy control C-rich aptamer (^18^F-hyCRO) via tail-vein injection and PET imaging was performed 60 min post-injection. ^18^F-hyAS1411 clearly showed that tumor-specific accumulation, comparing with the ^18^F-hyCRO.

The sgc8 is a 41-nt DNA aptamer that binds to protein tyrosine kinase 7 (PTK-7) [57], which is overexpressed in several human malignancies, was conjugated with ^18^F using click chemistry [58]. The ^18^F-tagged DNA aptamer sgc8 was injected into nude mice bearing HCT116 colon cancer tumors, which are positive for PTK-7. ^18^F-sgc8 clearly visualized the engrafted tumor region at 2 h post-injection. The main clearance route was reported as urinary and hepatic.

### 4.5. Computed Tomography (CT)

CT imaging is a commonly used technique to examine anatomical structures. However, contrast agents/probes for this modality are very limited. Recently GNPs have emerged as new nanoplatforms for CT imaging. As a proof of concept for CT imaging, anti-PSMA RNA aptamers were conjugated to gold nanoparticles and used as a CT contrast agent in PC [59]. The PSMA aptamer-conjugated gold nanoparticles showed more than 4-fold greater CT intensity for targeted LNCaP cells than that of nontargeted PC3 cells, but no in vivo imaging was carried out.

As another CT imaging approach, fluorescent gold nanoparticles were conjugated with diatrizoic acid and the anti-nucleolin AS1411 aptamer (AS1411-DA-AuNPs) [60]. The fluorescence spectrum of AS1411-DA-AuNPs showed a clear orange-red emission, with a maximum at 620 nm. In vivo molecular imaging was investigated in CLI-5 lung adenocarcinoma-bearing NOD-SCID mice via tail-vein injection of AS1411-DA-AuNPs. CT imaging was performed 30 min post-injection and showed a 106% more contrast enhancement in CLI-5 tumor-bearing mice than before-injection. In parallel, the orange-red fluorescence emitting from AS1411-DA-AuNPs in the CLI-5 tumor could also be visualized by the naked eye [60].

### 4.6. Ultrasound (US)

The use of gas-filled nanobubbles (NBs) constructed from poly (lactide-co-glycolic acid; PLGA), which is a biodegradable copolymer, improves US signals [61]. Therefore, it has been popularized as contrast agent for CT imaging. For targeted molecular imaging, the anti-PSMA A10-3.2 aptamer was conjugated with PLGA NBs (A10-3.2-PLGA NBs) [62]. For in vivo imaging, the A10-3.2-PLGA NBs were injected into LNCaP PC engrafted nude mice intravenously. Compared with saline control group, the distribution of contrast agent echo signals was rich and enhancement of contrast were observed in mice treated with A10-3.2-PLGA NBs [62]. The A10-3.2-PLGA NBs showed strong signal intensity, peaking at approximately 25 dB.

### 4.7. Photoacoustic (PA) Imaging

PA imaging has recently emerged as an alternative to MRI and X-ray tomography in the biomedical field, due to the ability to capture high resolution images at depth. However, the lack of selective probes makes its application in limit. For an experimental PA imaging by aptamers, a thrombin binding aptamer (TBA) was hybridized with IRDye 800CW-labeled (FDNA) and IRDye QC-1 quencher-labeled (QDNA) single-stranded DNA to form a DNA duplex structure for imaging probes [63]. When binding of target, it triggered the release of quencher strand and induced the relief the quenching strand. Thus, it induced the change of the PA signal at 780/725 nm. In other words, the binding of thrombin (TBA) loosed the hybridization of TBA-QDNA complexes and resulted in a release of FDNA, increase of both fluorescence and PA signals. Without thrombin, IRDye was placed next to quencher, induction of low fluorescence and PA signals.

In experimental mice imaging work, the DNA probes were injected into each flank of BALB/c mice. After then, thrombin and PBS were injected to each flank of mice. The PA images recorded at 45 min post-injection in thrombin injected mice showed a higher PA signal ratio at 780/725 than PBA or untreated mice.

### 4.8. Multimodal Imaging

Every molecular imaging modality has unique advantages and disadvantages. Therefore, current trend of imaging is to develop multimodal imaging to increase the diagnostic accuracy. As a proof of concept for a multimodal imaging system, multimodal nanoparticles have been developed for optical, MR, and PET imaging. For single multimodal nanoparticle imaging agent, cobalt ferrite magnetic nanoparticles surrounded by fluorescent rhodamine (termed MF) within a silica shell matrix were conjugated with anti-underglycosylated mucin-1 (uMUC-1) aptamer (MF-uMUC-1). Following step, MF-uMUC-1 was labeled by Gallium siotype ^68^Ga (termed MFR-uMUC-1) with a p-SCN-bn-NOTA chelating agent [64]. For in vivo imaging of aptamer based multimodal imaging, MFR-uMUC-1 nanoparticles or MFR-uMUC-1 mutant (uMUC-1 mt, control) nanoparticle were injected into BT-20 breast cancer cell implanted nude mice throughout tail vein.

Three imaging modalities; fluorescence, PET, and MRI were used to take images at multi-time points such as 2, 12, 24 h after IV injection. The PET images showed significant accumulation of ^68^Ga radioactivity in the tumor region of the MFR-uMUC-1-injected group, but not in the MFR-uMUC-1 mt-injected group. In the MFR-uMUC-1 injected group, significantly high tumor-specific fluorescence was also observed. By MR images, it showed significantly enhanced dark signals in MFR-uMUC-1-injected mice [64]. These results demonstrate a high specificity for targeting cancers using the uMUC-1 aptamer and suggest it is feasible as a single multimodal probe in vivo by aptamer guided.

**Figure 3:**
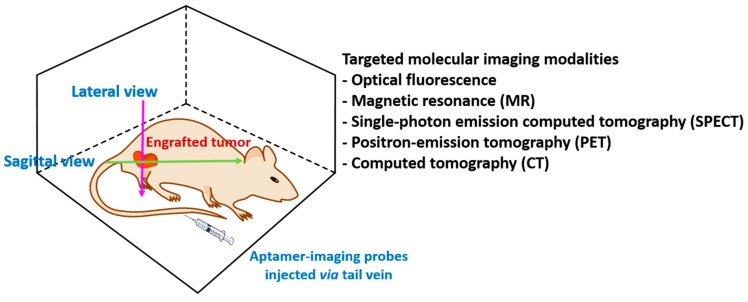
Targeted in vivo imaging using aptamers. Mice possessing xenografted cancer cells are injected with aptamers labeled with imaging dyes. Non-invasive imaging modalities such as fluorescence, magnetic resonance (MR), single-photon emission computed tomography (SPECT), position-emission tomography (PET), or computed tomography (CT) are used for imaging on lateral or sagittal view.

Taken together, targeted molecular in vivo imaging by aptamers as depicted in Figure 3 shows cancer-specific recognition in multiple imaging modalities. Each modality has various advantages and disadvantages. Fluorescence imaging is very cost effective and shows high sensitivity, but high autofluorescence and poor tissue penetration prevent the translation of fluorescence imaging into clinics. Nuclear imaging such as SPECT and PET provide improved resolution of imaging and longitudinal assessment of deep tissues in the same patient with high sensitivity, but the expensive cost and handling of isotypes are disadvantages. MRI and CT are excellent techniques to examine anatomical body structures, so enhancement of contrast with imaging agents is a key factor. Currently, aptamer-guided molecular imaging remains in the preclinical stage, but we expect that aptamer-guided molecular imaging can be used in clinics in the near future.

## 5. Clinical Trials

Clinical trials using aptamers for imaging or diagnostics are actively under investigation. For instance, the anti-PTK-7 DNA aptamer (^68^Ga-sgc8) is under interventional clinical trial, *ClinicalTrials Identifier: NCT03385148*. The aim of this study is to assess its safety and biodistribution, and to evaluate its application for PET/CT scan in colorectal patients. 10–20 mBq of ^68^Ga-Sgc8 will be injected into colorectal cancer patients. Diagnosis efficacy of ^68^Ga-Sgc8 PET/CT scans will be compared with those of ^18^F-FDG PET. Patients are currently being recruited for this study.

An aptamer-based biosensor to detect a biomarker in bladder cancer is under perspective observational clinical trial, *ClinicalTrials Identifier: NCT02957370*. The aim of this trial is to discover urinary biomarkers in bladder cancer patients using a Förster resonance energy transfer (FRET) system. Patients are currently being recruiting for this study.

## 6. Chemical Modifications

**Figure 4:**
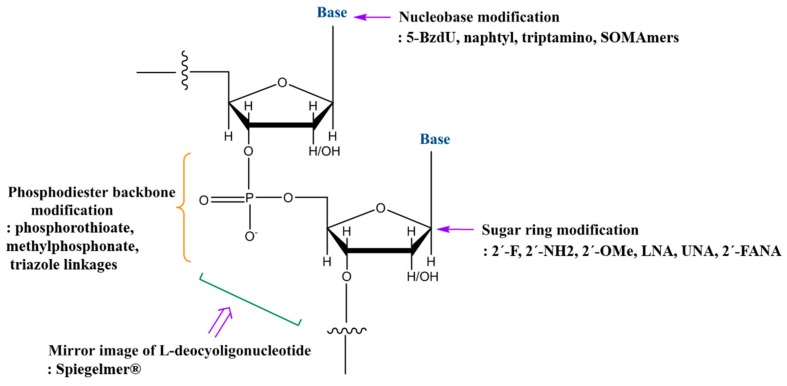
Strategies for chemical modification of nucleic acid aptamers to increase structural stability and nuclease resistance. The most commonly used strategies in DNA/RNA aptamers are modifications of the phosphodiester linkage, the sugar ring, and the bases.

To facilitate the translation of aptamer-guided molecular imaging, chemical modifications should be considered to increase resistance to cellular nucleases and to improve structural stability and binding affinity [65]. The most common strategies employed are depicted in Figure 4. This includes: phosphodiester backbone modifications such as phosphorothioate, methylphosphonate, and triazole linkages; base modifications such as 5-(*N*-benzycarboxyamide)-2′-deoxyuridine (5-BzdU), naphtyl, triptamino, and isobutyl in Slow Off-rate Modified aptamers (SOMAmers); sugar ring modification such as 2′-fluoro (2′-F), 2′-amino (2′-NH2), 2′-O-methyl (2′-OMe), locked nucleic acid (LNA), unlocked nucleic acid (UNA), 2′-deoxy-2′-fluoro-D-arabinonucleic acid (2′-FANA); the mirror image of l-deoxynucleotides in Spiegelmer^®^ [65]. Such chemical modifications are mainly developed for therapeutic purposes to avoid immune responses and to increase serum resistance and potency. To date, DNA aptamers and 2′-F RNA aptamers have been proven as targeted imaging probes in tissue and in vivo imaging. We believe that introducing these chemical modifications improves the function of aptamers as targeted imaging probes, because improvement of nuclease resistance and structural stability will increase the retention time of aptamers in the body and target specificity. Indeed, the efficacy of anti-EpCAM and nucleolin LNA modified aptamer conjugated with Fe_3_O_4_ nanoparticles (NPs) was tested for multimodal imaging for NIR, MRI, and CT imaging in vivo [66]. After feeding the formula for 48 h, imaging was taken by three imaging modalities. Comparing with untreated control, comparatively higher fluorescence was shown by NIR imaging. Also, comparatively high contrast in tumor region was observed by NIR and CT. It suggests that chemical modifications of aptamer are well tolerated as imaging probes. On the other hand, manufacturing costs will be increased.

## 7. Conclusions and Implications for Clinical Use

Currently, targeted molecular imaging using aptamers is an actively developing research field. For targeted imaging modalities, aptamers have shown cancer-specific recognition in tissue imaging and in vivo imaging in multiple cancer models.

For in vivo use, DNA aptamers (anti-nucleolin, anti-MUC1, anti-EGFRvIII, anti-cancer cell-type-specific aptamers) and RNA aptamers (anti-PSMA aptamer) show perform well for cancer-specific detection using various imaging techniques (e.g., fluorescence, PET, SPECT, MRI, CT, and US) at the preclinical stage. This suggests that aptamers have great potential for targeted imaging agents to differentiate disease-specific targets, which will be applicable in the clinic. As described in Section 4, every imaging technique has its own detecting mechanism, and unique pros and cons. Thus, a multimodal imaging platform might be a preferred option to fill the gap. Indeed, SPECT-CT and PET-CT are currently standard in most clinics. As for aptamer-guided multimodal imaging, optical-MR-PET imaging techniques were investigated with anti-MUC-1 aptamers [64], which showed high cancer-specific detection. Thus, construction of new multimodal imaging modalities that incorporate aptamers, such as PET-MRI, SPECT-MRI, fluorescence-PET, and US-PET, will play a leading role in developing novel, clinically relevant approaches. Regardless of what type of imaging techniques will be used, targeted molecular imaging by aptamers shows quick accumulation into target sites that gives suitable target-to-background signals. Thus, we believe that aptamer-guided imaging will improve diagnostic accuracy.

The next promising aptamer-guided imaging application to be developed is tissue MSI imaging. Aptamers conjugated with gold metal already demonstrated discrimination between cancer and normal tissues [27], suggesting that aptamers are suitable molecular probes in MSI. For the analysis of multiple biomolecules on single specimens, conjugation of multiple cancer-specific aptamers with different metals will be developed in future strategies, which can be utilized to characterize clinical human cancer specimens.
